# Evaluation of the blended public health empowerment program-basic field epidemiology in the Eastern Mediterranean Region

**DOI:** 10.3389/fmed.2024.1391219

**Published:** 2024-07-15

**Authors:** Ruba Kamal Alsouri, Yousef Khader, Haitham Bashier, Mirwais Amiri, Sara Abdelkarim Morsy, Zainab Naseer Abbas, Zeina Elias Farah, Mohannad Al Nsour

**Affiliations:** ^1^Global Health Development, Eastern Mediterranean Public Health Network, Amman, Jordan; ^2^Department of Community Medicine, Public Health, and Family Medicine/Faculty of Medicine, Jordan University of Science and Technology, Ar-Ramtha, Jordan; ^3^Field Epidemiology Training Program (FETP) Department, Egypt Ministries of Health (MOH), Minya, Egypt; ^4^Department of Epidemiology, Public Health Directorate, Ministry of Health, Baghdad, Iraq; ^5^Lebanese Field Epidemiology Training Program, Epidemiological Surveillance Program, Beirut, Lebanon

**Keywords:** field epidemiology training program, blended learning, frontline FETP, evaluation, health workforce development

## Abstract

**Introduction:**

The COVID-19 pandemic encouraged the shift toward technology-based learning globally, impacting education systems profoundly. In response to this emerging need, the Eastern Mediterranean Public Health Network (EMPHNET) adapted its Public Health Empowerment Program-Basic Field Epidemiology (PHEP-BFE) to a Blended Learning Model. This study evaluates the Blended PHEP-BFE program in Iraq, Egypt, and Lebanon, focusing on participant reactions and learning outcomes.

**Methods:**

A descriptive evaluation was conducted, aligned with the first two levels of Kirkpatrick's model. Online questionnaires were administered to participants and facilitators through EMPHNET's Learning Management System (LMS). Qualitative and quantitative data were analyzed to assess program effectiveness, satisfaction, and challenges.

**Results:**

A total of 138 PHEP-BFE participants (119 (86.2%) males and 19 (13.8%) females) from Iraq (*n* = 61), Egypt (*n* = 66), and Lebanon (*n* = 11) responded to the questionnaire. The majority of the participants (96.4%) reported that they were satisfied with PHEP-BFE. Notably, 77.5% of participants rated the blended learning program as very good or excellent, 18.1% rated it good, and 3.6% found it average, with a minimal 0.7% expressing dissatisfaction. The majority of participants agreed that the blended PHEP-BFE enhanced their capacity to conduct, review and monitor surveillance data (95.7%), perform descriptive data analysis (94.2%), effectively communicate information with agency staff and the local community (95.7%), write summaries of surveillance findings or outbreak investigations (95.7%), use MS Excel to enter, analyze, and display public health surveillance data (91.3%), prepare and administer an oral presentation for fieldwork (94.9%), and increase their knowledge of fundamental field epidemiology (94.9%). The participants responded positively to the program's content, training duration, learning platform, facilitators and mentors, and fieldwork.

**Conclusion:**

The study showcases the success of the blended PHEP-BFE in diverse contexts, emphasizing positive participant reactions and improved competencies. The evaluation underscores the program's success in advancing public health training in the EMR. Blended learning models prove promising for future FETP initiatives, contributing valuable insights to public health workforce development. Positive outcomes and identified challenges, provide a roadmap for continuous improvement.

## Introduction

Traditional pedagogical models have recently changed toward a greater use of technology that supports knowledge delivery and acquisition. In light of the COVID-19 pandemic, public health and education systems across the globe were gravely affected. According to the United Nations (UN), 94% of the world's student population was impacted, involving more than one billion learners in more than 190 countries (1). Due to the virulent spread of COVID-19, many countries switched from face-to-face learning to remote/virtual learning. E-learning plays a crucial role in education, and it is now a popular modality (2). Many users of e-learning platforms agree that online education makes it easier for e-learning to be administered and for learners to access instructors and learning resources (3).

Since its establishment in 2009, the Eastern Mediterranean Public Health Network (EMPHNET) has developed and strengthened Field Epidemiology Training Programs (FETPs) in the Eastern Mediterranean Region (EMR). FETPs, founded and supported by the U.S. Centers for Disease Control and Prevention (CDC) in 1980, are competency-based training programs customized to country contexts and designed to build the global health workforce of field epidemiologists to strengthen surveillance systems and respond to health threats (4–6). Ministries of Health (MOHs) and other public health departments are supported and assisted by the CDC and its partner organizations in establishing FETPs in their nations (7). Since its inception, the program has been instrumental in strengthening the rapid response teams' roles within the MoHs, especially in public health directorates with FETP graduates. FETPs are crucial to achieving global health security because part of achieving the highest level of global public health security is advancing capacity building within the public health infrastructure of all countries (8) to lead local public health initiatives and support national public health systems (9).

**Figure 1:**
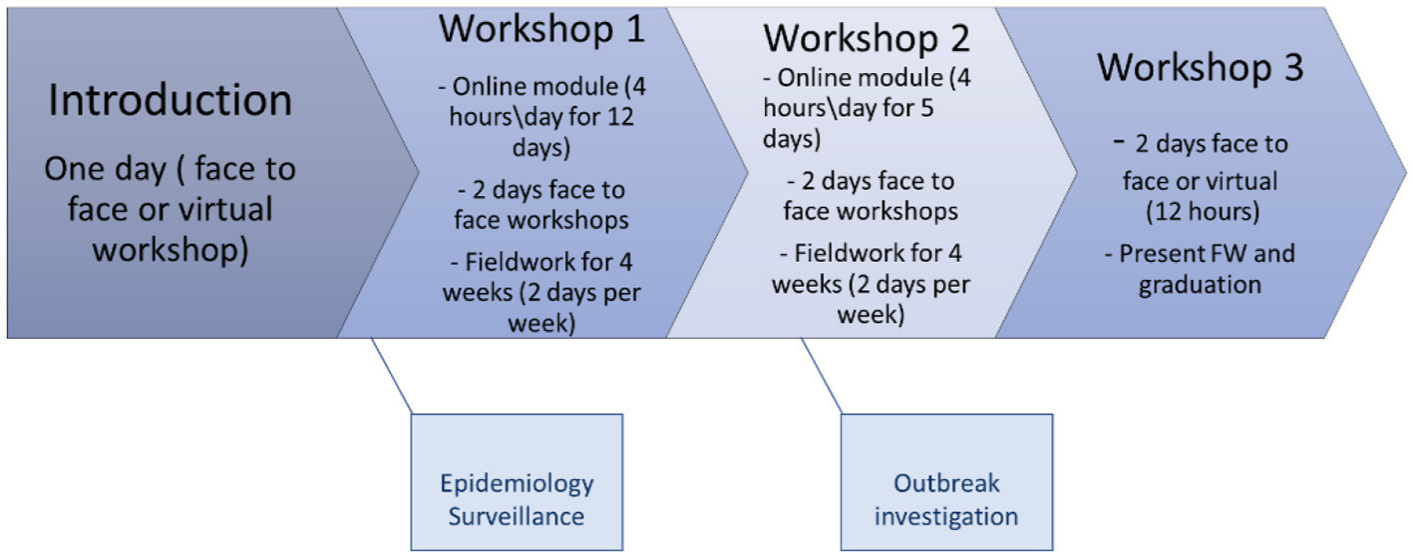
An illustration of blended PHEP-BFE structure.

FETPs are architecture to fit a three-tiered pyramidal model built of a 3-month modality (Basic/Frontline), a 1-year modality (Intermediate), and a 2-year modality (Advanced). The traditional FETP model involves 100% face-to-face learning. Public Health Empowerment Program- Basic Field Epidemiology (PHEP-BFE) is a 3-month program that focuses on the detection of and response to diseases and events of national and international public health concern. Trainees learn and practice fundamental skills used in surveillance, outbreak investigation, and basic management. EMPHNET has leveraged technology in its FETP training by developing its online learning portal, the Learning Management System (LMS), and offering FETPs in the region (10, 11). With the support of EMPHNET, countries including Iraq, Egypt, Lebanon, Morocco, Sudan, and Tunisia they adapted their basic and intermediate level-FETPs to the blended learning modality. Blended learning combines the most significant aspects of both traditional classroom instruction and non-traditional learning environments to give students the best of both worlds (12–14). Blended learning is a hybrid between synchronous learning and asynchronous self-paced learning. The blended PHEP-BFE program consists of two asynchronous, self-paced learning modules, three synchronous instructor-led workshops, and two on-the-job fieldworks as illustrated in Figure 1.

Various FETPs in the EMR implemented the e-learning system and pivoted to blended learning when traditional learning methodologies were impossible to implement, were too risky, or less cost-effective. During the past 2 years, FETPs adopted the blended modality in implementing the PHEP-BFE and graduated four cohorts in Iraq, two in Egypt, and one in Lebanon.

Among the various evaluation frameworks that are available, the Kirkpatrick evaluation model allows for the measurement of training effects at four levels: Level 1 (Reaction), Level 2 (Learning), Level 3 (Behavior), and Level 4 (Results) (15). Each level of the evaluation chronology is conducted over a period that ranges from the start of the program to 1–2 years post-program completion (15). Evaluating the blended PHEP-BFE is critical to measure the impact and explore the training program's efficacy in achieving program objectives, meeting target competencies, and supporting high-quality FETP training, including how each element contributes to achieving the desired results (15, 16). This study aimed to evaluate the blended PHEP-BFE in Iraq, Egypt, and Lebanon. Specifically, the main objectives of the study were to assess the LMS (17) in terms of ease of use and appearance, assess the level of satisfaction of participants and instructors with the program and with the learning material, evaluate the knowledge gained and the change in work performance, and identify challenges. Evaluation results will not only determine the potential of blended-learning FETP programs. Still, they will also assess whether this learning methodology can pave a promising path for future FETP-training programs. Findings from this evaluation study will inform evidence-based decisions on how to improve, maintain, and achieve high-quality programs for different FETPs in the region. They will provide information to help other countries adopt the blended modality.

## Methods

### Study design

A descriptive evaluation study was conducted from August 2021 to August 2022. The evaluation process followed the first two levels of the Kirkpatrick model framework, namely Level 1 (Reaction) and Level 2 (Learning) (15). These two levels of the mentioned evaluation framework were conducted sequentially, with Level 1 (Reaction) throughout the training from its onset till the end and Level 2 (Learning) periodically as spot checks during the training sessions, plus a formal assessment at the end.

A more formal evaluation of Level 1 was implemented immediately at the end of the training program and evaluated “reaction” by targeting learners, facilitators, and mentors. The purpose of level 1 evaluation was to answer “How did participants react to the training (e.g., satisfaction level)?”. Level 2 evaluated “learning” by targeting learners and mentors. The purpose of the level 2 evaluation was to answer, “To what extent did participants improve knowledge and skills and change attitudes as a result of the training?” Participants completed the online questionnaire that was distributed through EMPHNET LMS (17), and the facilitators and organizers completed the online questionnaire that was distributed through Crowdsignal (formerly PollDaddy) (18).

The study included all participants who completed the PHEP-BFE during the specified study period from Iraq, Egypt, and Lebanon which implemented the program from the EMR. This approach allowed for a comprehensive analysis of the program's effectiveness across diverse health care settings within the region, offering insights into both country-specific and regional impacts. The participants for the program were carefully chosen through nominations by their respective health departments, ensuring that all selected individuals were actively engaged in public health roles suitable for advanced training in field epidemiology. This selection process was designed to harness the expertise of health professionals who could directly apply and disseminate the learned skills within their local health systems.

### Questionnaires

To ensure a holistic understanding and continuous improvement of the PHEP-BFE Blended Learning Program, separate assessment tools looked at key perspectives from participants, facilitators, and organizers. The participants' evaluation questionnaire (Supplementary material) collected information on demographic characteristics, overall satisfaction, content organization, and the effectiveness of learning methods. The Likert scale questions provided a nuanced understanding of participants' views on specific program aspects, including competencies and skills development, facilitators, and the learning platform. Open-ended questions offered qualitative insights, allowing participants to express preferences, challenges, and suggestions for improvement, contributing to ongoing program enhancement aligned with participants' needs. The facilitator's evaluation focused on obtaining feedback from facilitators with diverse backgrounds. Their insights on various training aspects, including online sessions, collaboration, and the learning management system, were sought. Specific queries on the LMS platform's usability and facilitators' preferences ensured a thorough understanding of their experiences, enabling refinements to optimize the overall online learning experience. The tool for organizer's feedback was intended to capture the perspectives of organizers and support teams. Their evaluation encompassed participant and completion rates, facilitator commitment, training content, and the learning environment. Questions delving into participant and facilitator selection, program structure, and the LMS's effectiveness provided valuable insights. Feedback on challenges, achievement of objectives, and recommendations for future training informed crucial refinements for subsequent program iterations. All three assessment tools are available as Supplementary material annexed to this paper.

### Data analysis

Quantitative data were summarized using descriptive analysis using the IBM SPSS (version 24). Categorical variables were summarized as frequencies with percentages, *n* (%). As for qualitative data, a thematic analysis was performed to look for patterns of meaning in the dataset. The responses to each open-ended question were first reviewed to determine the overall sentiment, themes, and keywords. Individual responses were then coded with themes related to the corresponding questions. The themes' frequencies and percentages were then calculated for each question. For learning evaluation, pre-and post-test results were analyzed using paired *t*-test to assess the changes in knowledge of participants post-completing the program compared to baseline. A *p*-value < 0.05 was considered statistically significant.

## Results

### Participants' characteristics

A total of 138 PHEP-BFE participants (119 (86.2%) males and 19 (13.8%) females) from Iraq (*n* = 61), Egypt (*n* = 66), and Lebanon (*n* = 11) responded to the questionnaire. More than half of the participants (57.2%) were public health officers. Most participants (91.3%) were selected to enroll in the program through direct nominations from their work supervisors. A total of 127 (92.0%) residents reported that they had never attended blended learning. The participants' characteristics are shown in Table 1.

**Table 1:** The characteristics of 138 participants who evaluated the Blended Public Health Empowerment Program- Basic Field Epidemiology (PHEP-BFE).

**Variable**	***n***	**%**
**Sex**		
Male	119	86.2
Female	19	13.8
**Country**		
Egypt	66	47.8
Iraq	61	44.2
Lebanon	11	8.0
**Selection criteria for enrollment in the program**		
Direct nomination by work supervisors	126	91.3
Direct application to the program	12	8.6
**Profession**		
Physician	6	4.3
Public health officer	79	57.2
Health sciences (pharmacy, nursing, other health professions)	41	29.7
Non-health professions	12	8.7

### Overall assessment of the blended PHEP-BFE

The majority of the participants (96.4%) reported that they were satisfied with PHEP-BFE. Notably, 44.9% of participants rated the blended learning program as excellent, while 32.6% considered it very good, 18.1% rated it good, and 3.6% found it average, with a minimal 0.7% expressing dissatisfaction. An impressive 96.3% of participants acknowledged the program's positive impact on their understanding of public health surveillance, outbreak investigation concepts, data analysis, interpretation skills, and fieldwork. Additionally, a substantial 90.6% of participants agreed that they had ample opportunities to comprehend and practice the acquired knowledge. The endorsement of the program was resounding, as 99.3% of participants expressed their willingness to recommend the PHEP-BFE program to their colleagues.

Table 2 shows the proportion of PHEP-BFE participants who agreed or strongly agreed with the evaluation items assessing various facets of the PHEP-BFE.

**Table 2:** The proportion of PHEP-BFE participants who expressed agreement or strong agreement with the evaluation items assessing various facets of the PHEP-BFE.

**Domains and items**	***n***	**%**
**Topic content: organization, relevance of the subjects, clarity of the materials**		
The program content was well organized and easy to understand	128	92.8
The program instructions were sufficient, helpful, and clear	127	92.7
The included case studies helped me understand concepts of surveillance and outbreak investigation clearly	134	97.1
The assignments complemented my understanding of the online sessions effectively	121	87.7
The material presented in the course was new to me	121	87.7
The material presented in the course was informative	133	96.4
The material presented in the course is applicable to my work setting	129	93.5
The material presented in the course is applicable to your professional development	132	95.7
The course content balanced between theoretical and practical	124	89.9
**Competences and skills development**		
The program developed my skills to conduct, review and monitor surveillance data collection	132	95.7
The program increased my knowledge in basic field epidemiology	131	94.9
The program developed my skills to perform descriptive data analysis	130	94.2
The program developed my skills to communicate information effectively with agency staff and with the local community	132	95.7
The program developed my skills to respond effectively to public health events, specifically, disease outbreaks	132	95.7
The program developed my skills to write a summary report on surveillance findings or an outbreak investigation	132	95.7
The program developed my skills to use Microsoft Excel to enter, analyze, and display public health surveillance data	126	91.3
The program developed my skills to prepare and administer an oral presentation of field work	131	94.9
**Program facilitators**		
The facilitators were good communicator	136	98.6
The facilitators were knowledgeable, had academic credibility and well prepared	133	96.4
The facilitators responded to my inquires in timely manner	134	97.1
The facilitators encouraged me to complete the program	135	97.8
The facilitators returned assignments in timely manner	132	95.7
The facilitators provided helpful feedback	129	93.5
**Mentors**		
The mentors were good communicator	131	94.9
The mentors responded to my inquires in timely manner	129	93.5
The mentors encouraged me to complete the program	129	93.5
**Training schedule**		
The program was well paced within the allotted time	112	81.2
The time allotted for each Module was appropriate	107	77.5
The daily training hours were satisfactory	113	81.9
The time allotted for the fieldwork assignments was appropriate	112	81.2
**Course overall**		
I was given an adequate opportunity to comprehend and practice what I was learning	125	90.6
I am satisfied with this course	133	96.4
**Learning platform**		
The platform was easy to access and use	129	93.5
The interface of the platform is user friendly	129	93.5
The appearance of the platform is attractive	125	90.6

### Program's content

The understandable content participant feedback regarding the program indicated a high level of satisfaction, with 92.8% attesting to program content's well-organized and easily comprehensible nature. Furthermore, an overwhelming majority (92.7%) expressed satisfaction with the adequacy, helpfulness, and clarity of the program instructions. A significant portion of participants, amounting to 87.7%, found the course material to be both new and very informative (96.4%), with applicability noted to their work setting (93.5%) and professional development (95.7%). The balance between theoretical and practical content was perceived positively by 89.9% of participants. Additionally, 87.7% reported that assignments effectively complemented their understanding of the online sessions.

The incorporation of case studies proved highly beneficial, with almost 97.1% of participants stating that they helped grasp the concepts of epidemiological surveillance and outbreak investigation. A substantial 92.1% credited the online sessions with enhancing their scientific and practical skills. Participants praised the flexibility of online meetings, citing the advantage of scheduling at convenient times and eliminating the need for travel.

However, 23.2% of participants suggested improvements in the quality of case studies. Additionally, 14.5% recommended more exercises in using Excel and computer skills for epidemiology. Concerns about session duration were raised, with almost 38% feeling that training sessions and workshops were too short and 34% suggesting an increase in training sessions. Furthermore, one participant suggested providing downloadable lecture content for convenient access.

### Competencies and skills development

The majority of participants agreed that the blended PHEP-BFE enhanced their capacity to conduct, review and monitor surveillance data (95.7%), perform descriptive data analysis (94.2%), effectively communicate information with agency staff and the local community (95.7%), write summaries of surveillance findings or outbreak investigations (95.7%), use MS Excel to enter, analyze, and display public health surveillance data (91.3%), prepare and administer an oral presentation for fieldwork (94.9%), and increase their knowledge of fundamental field epidemiology (94.9%).

### Training duration

The program was running at an appropriate pace for 81.2% of the participants. The time allotted for each module and the assignments given during the implementation were appropriate for 77.5% and 81.2% (*N* = 112), respectively. Furthermore, the daily training hours were adequate for 81.9%, and the online sessions' duration was reasonable for 68.8%. However, around 11.6% claimed that listening to an online lecture for more than 2 h a day was long.

### Learning platform

The educational platform was easy to use, and the interface was user-friendly for 93.5% of the participants. Moreover, the platform interface was attractive for the learning experience of 90.6%. The majority of participants evaluated different learning methods as good, including videos and online self-paced sessions (87.7%), group work and discussions (92%), assignments (92.8%), case studies (95.7%), and face-to-face workshops (92.8%).

### Facilitators and mentors

Almost 96.4% of participants reported that the facilitators had credible academic and cognitive competencies and were well-prepared. Most reported that facilitators were good communicators (98.6%) and encouraged the participants to complete the program (97.8%). Also, 97.1% of participants reported that the facilitators responded to their inquiries promptly, and 93.5% provided helpful feedback. The mentors communicated very well with the participants, as mentioned by 94.9% of respondents, encouraged participants, and answered their questions promptly (93.5%).

### Fieldwork

Nearly 97.8% of participants across the three countries affirmed that the fieldwork in the program met their expectations. Furthermore, a substantial 60.9% of participants acknowledged the adequate preparation of the fieldwork. However, challenges were encountered implementingfieldwork during fieldwork in the implementation of the PHEP-BFE program in these countries. Transportation issues were notable, with 17.4% of participants facing difficulties due to the considerable geographical distance between workstations and the field. An additional 14.5% reported experiencing delays in completing online sessions as scheduled due to poor internet connections. Moreover, 13.7% could not fulfill program assignments due to competing work engagements.

Approximately 10.8% of participants highlighted concerns about data inaccuracy, emphasizing the need to enhance databases in their respective countries. An expressed desire for more practical experience and additional fieldwork was voiced by 14.5% of participants. Additionally, 8.7% requested more case studies to bolster their epidemiological skills. While fieldwork activities were deemed relevant by most participants (73.9%), a noteworthy 8.7% reported that the content was entirely new and not aligned with their typical work experiences.

### Facilitators' reaction

Eight facilitators from the three countries completed the questionnaire at the conclusion of the training. Seven facilitators were satisfied with the overall training program in terms of training modality, content, and platform. Seven facilitators rated the online sessions of good to very good, stating that they were informative, contextualized, and well organized. *A*ll facilitators agreed that the LMS platform is simple to use and navigate and user-friendly.

## Discussion

FETP has consistently illustrated its significant influence on enhancing field epidemiology capacities across various countries (19–24). However, this is the only study that aimed to evaluate the blended modality of the program using Level 1 (Reaction) and Level 2 (Learning) aspects of the Kirkpatrick model (15).

Our evaluation showed the effectiveness of the blended PHEP-BFE in the three countries, which provide a relatively wide representation of the region due to their varying contexts. It highlights how the blended approach successfully enabled participants to develop their skills and abilities in data analysis, writing reports, epidemiology surveillance, conducting studies, outbreak investigation, and response. Participants had an overall positive reaction to the approach, including modalities format, effectiveness, modalities, and platform.

The findings from the participants in the PHEP-BFE program are overwhelmingly positive, reflecting high levels of satisfaction and perceived effectiveness. An impressive 96.4% of participants expressed satisfaction with the PHEP-BFE program. This high satisfaction rate suggests that the program meets or exceeds the expectations of most participants. The blended learning approach received favorable ratings from participants, indicating a robust positive perception of the program's delivery and content. Almost the entire participant cohort, at 96.3%, acknowledged the positive impact of the program on their understanding of public health surveillance, outbreak investigation concepts, data analysis, interpretation skills, and fieldwork. This suggests that the program is effectively fulfilling its educational objectives. The resounding endorsement of the program is evident in the fact that 99.3% of participants expressed their willingness to recommend the PHEP-BFE program to their colleagues. This high recommendation rate signifies participant satisfaction and confidence in the program's value and effectiveness.

The findings regarding the program's content reflect a generally high level of satisfaction among participants. Participants praised the flexibility of online meetings, citing the advantage of scheduling at convenient times and eliminating the need for travel. Notably, 23.2% of participants suggested improvements in the quality of case studies, indicating a desire for enhanced depth or relevance. About 14.5% recommended more exercises on using Excel and computer skills for epidemiology, suggesting a need for practical skill-building. Concerns about session duration (38%) and the number of training sessions (34%) were raised, suggesting a desire for more comprehensive and in-depth training experiences. The suggestion for downloadable lecture content reflects a need for flexibility in accessing materials, indicating a consideration for participants' convenience. Addressing these suggestions could enhance the overall participant experience and the program's effectiveness.

### Top of form

The findings indicate that the blended PHEP-BFE program has significantly contributed to the enhancement of participants' skills and capacities in various areas, with an overwhelmingly high percentage of agreement across different competencies. Notable areas of improvement include conducting, reviewing, and monitoring surveillance data, performing descriptive data analysis, effectively communicating information, writing summaries of surveillance findings or outbreak investigations, using MS Excel, and preparing and administering oral presentations for fieldwork. These results underscore the program's success in equipping participants with practical and applicable skills. The majority of participants found the program's overall pace to be appropriate, suggesting that the learning modules and assignments were well-structured. The fact that 81.2% found the duration of assignments appropriate further supports the program's well-designed curriculum. While the majority found the daily training hours and online sessions suitable, 11.6% expressed concern about the duration of listening to an online lecture for more than 2 h a day, indicating potential fatigue or challenges with extended online engagement.

Participants appreciated the learning platform, praised the facilitators and mentors for their competence and communication skills, and reported high satisfaction with the fieldwork component. Nevertheless, participants faced challenges during fieldwork, most notably lacking access to accurate data. Also, multiple challenges to implementing the blended approach were revealed, including the negative effect of the weak telecommunications infrastructure, such as in Lebanon and Iraq, and the digital divide between urban and rural areas. Other challenges were the lack of adequate time, which was reported by some residents who faced difficulties completing the program given their preoccupation with their jobs. However, this challenge affects both the online and the face-to-face aspects of the program, and is not unique to the blended approach.

Feedback from facilitators indicated high satisfaction with the overall training program, including training modality, content, and the learning management system (LMS) platform. Ratings of online sessions, as good to very good, emphasizing informativeness, contextualization, and organization, showcase the effectiveness of the training content. The unanimous agreement among facilitators that the LMS platform is simple, user-friendly, and easy to navigate further supports the success of the learning environment.

The online modality was beneficial to participants as no travel was required. The result of the program was predicated on the idea that enhancing the epidemiologic expertise of MOH employees will increase the MOH's ability to identify, prevent, and address public health priority concerns, ultimately enhancing the general population's health (25). A key indicator of the quality and sustainability of an FETP is a career path created by the MoH or other organizations where the graduates will workto draw in and keep the finest and brightest individuals (6).

While the study provides valuable insights into the success and positive outcomes of the blended PHEP-BFE in Iraq, Egypt, and Lebanon, it is important to acknowledge some potential limitations. The study's participant pool may not entirely represent all PHEP-BFE participants in the Eastern Mediterranean region. Participants who chose to respond to the online questionnaires may differ systematically from those who did not, introducing potential sampling bias. The study focuses on participant reactions and immediate learning outcomes, providing a snapshot of the program's success. Long-term impacts and sustainability of the acquired skills over time are not assessed, limiting the understanding of the program's enduring effects.

While the results of this evaluation are promising, they must be considered in the context of potential biases and external influencing factors. The reliance on self-reported data may introduce bias, as participants could overestimate their satisfaction or learning outcomes. External factors including various levels of support from local health departments and differing access to technology among participants could also have influenced the outcomes.

### Limitations

This study's findings are subject to several limitations. Geographical and demographic constraints may limit the generalizability of the findings to other regions or populations within the Eastern Mediterranean. The study encompassed participants from only three countries, which may not represent the entire diversity of public health contexts in the region. Additionally, the study period coincided with the ongoing COVID-19 pandemic, which might have affected participants' availability and engagement due to heightened professional demands.

The study focuses on Reaction and Learning, and a longer-term impact would be necessary. The evaluation was conducted over a relatively short period (one year). Longer-term studies are needed to assess the sustained impact of the training on public health practices.

## Conclusion

This study showcases the success of the blended PHEP-BFE in diverse contexts, emphasizing positive participant reactions and improved competencies. The evaluation underscores the program's success in advancing public health training in the EMR. Blended learning models prove promising for future FETP initiatives, contributing valuable insights to public health workforce development. Blended public health training programs can be as effective as traditional modalities if their pedagogical design is carefully planned considering the countries' context, infrastructure, and capacities, this is coupled with identified challenges but will provide a roadmap for continuous improvement. There is a need to evaluate the impact of the blended program on the service provision.

## Data availability statement

The original contributions presented in the study are included in the article/supplementary material, further inquiries can be directed to the corresponding author.

## Ethics statement

Ethical approval was not required for the study involving humans in accordance with the local legislation and institutional requirements. The studies were conducted in accordance with the local legislation and institutional requirements. Written informed consent to participate in this study was not required from the participants in accordance with the national legislation and the institutional requirements.

## Author contributions

RA: Conceptualization, Investigation, Writing – original draft, Writing – review & editing. YK: Formal analysis, Writing – review & editing. HB: Supervision, Writing – review & editing. MA: Writing – review & editing. SM: Writing – review & editing. ZA: Writing – review & editing. ZF: Writing – review & editing. MA: Supervision, Writing – review & editing.
