# Supplementary material for: Evaluation of the blended public health empowerment program-basic field epidemiology in the Eastern Mediterranean Region
Source: Front Med (Lausanne). 2024 Jul 15;11:1391219. doi: 10.3389/fmed.2024.1391219 (PMC11284125; doi:10.3389/fmed.2024.1391219)
Supplement: Supplementary file 1 [file Table_1.DOCX]

**Supplementary file: Evaluation questionnaire**

**Q1. Gender:**

|  | Male |
| --- | --- |

|  | Female |
| --- | --- |

**Q2. Age:**

**Q3. Country:**

**Q4. How were you selected to participate in this program(s)? (Please tick one).**

- Through open competition
- Direct nomination by work supervisor\s
- Direct application to the program
- Other

If Other, please specify:

**Q5. Which of the following best describes your professional discipline?** (Please put a cross in one or more boxes)

- Medical doctor
- Public health officer
- Health Sciences (medicine, nursing, other health professions)
- Other non-health professions – (please specify)

If Other, please specify:

**Q6.** Is this your first time participating in a training held by GHD|EMPHNET?

- Yes
- No

**Q7.** Is this your first time participating in a Blended learning program?

- Yes
- No

**Q8.** What is your overall assessment of the PHEP-BFE Blended Learning Program?

- Excellent
- Very Good
- Good
- Average
- Poor
- Very Poor

Please help us understand why you selected this answer.

**Q9. Please rate the following:**

|  | Strongly disagree | disagree | Neural | Agree | Strongly agree | |
| --- | --- | --- | --- | --- | --- | --- |
|  | 1 | 2 | 3 | 4 | | 5 |
| **9.1 Topic Content: Organization, Relevance of the subjects, Clarity of the Materials** | | | | | | |
| 9.1.1 The program content was well organized and easy to understand |  |  |  |  |  | |
| The program instructions were sufficient, helpful, and clear |  |  |  |  |  | |
| The included case studies helped me understand concepts of surveillance and outbreak investigation clearly |  |  |  |  |  | |
| The assignments complemented my understanding of the online sessions effectively. |  |  |  |  |  | |
| The material presented in the course was new to me |  |  |  |  |  | |
| The material presented in the course was informative. |  |  |  |  |  | |
| The material presented in the course is applicable to my work setting |  |  |  |  |  | |
| The material presented in the course is applicable to your professional development. |  |  |  |  |  | |
| The course content balanced between theoretical and practical |  |  |  |  |  | |
| **Competences & Skills Development** | | | | | | |
| The program developed my skills to conduct, review and monitor surveillance data collection |  |  |  |  |  | |
| The program increased my knowledge in basic field epidemiology. |  |  |  |  |  | |
| The program developed my skills to perform descriptive data analysis |  |  |  |  |  | |
| The program developed my skills to communicate information effectively with agency staff and with the local community |  |  |  |  |  | |
| The program developed my skills to respond effectively to public health events, specifically, disease outbreaks |  |  |  |  |  | |
| The program developed my skills to write a summary report on surveillance findings or an outbreak investigation |  |  |  |  |  | |
| The program developed my skills to use Microsoft Excel to enter, analyze, and display public health surveillance data |  |  |  |  |  | |
| The program developed my skills to prepare and administer an oral presentation of field work |  |  |  |  |  | |
| **Program Facilitators** | | | | | | |
| The facilitators were good communicator |  |  |  |  |  | |
| The facilitators were knowledgeable, had academic credibility and well prepared. |  |  |  |  |  | |
| The facilitators responded to my inquires in timely manner. |  |  |  |  |  | |
| The facilitators encouraged me to complete the program |  |  |  |  |  | |
| The facilitators returned assignments in timely manner. |  |  |  |  |  | |
| The facilitators provided helpful feedback |  |  |  |  |  | |
| **Mentors** | | | | | | |
| The mentors were good communicator |  |  |  |  |  | |
| The mentors responded to my inquires in timely manner. |  |  |  |  |  | |
| The mentors encouraged me to complete the program |  |  |  |  |  | |
| **Training schedule** | | | | | | |
| The program was well paced within the allotted time |  |  |  |  |  | |
| The time allotted for each Module was appropriate |  |  |  |  |  | |
| The daily training hours were satisfactory |  |  |  |  |  | |
| The time allotted for the fieldwork assignments was appropriate |  |  |  |  |  | |
| **Course Overall** | | | | | | |
| I was given an adequate opportunity to comprehend and practice what I was learning. |  |  |  |  |  | |
| I am satisfied with this course |  |  |  |  |  | |
| **Learning platform** | | | | | | |
| The platform was easy to access and use |  |  |  |  |  | |
| The interface of the platform is user friendly |  |  |  |  |  | |
| The appearance of the platform is attractive |  |  |  |  |  | |

**Q10.** The length of the online sessions was:

- Long
- Neither Long nor Short
- Short

Please help us understand why you selected this answer.

**Q11.** How satisfied were you with the material presented at the online sessions?

- Very Satisfied
- Satisfied
- Neutral
- Dissatisfied
- Very Dissatisfied

Please help us understand why you selected this answer.

**Q12.** What did you like best about this program?

**Q13.** What did you like least about this program?

**Q14.** How could this program be improved?

**Q15.** Would you recommend this program to your colleagues?

- Yes
- No
- Please help us understand why you selected this answer.

**Q16.** How do you rate the below learning methods used?

| Videos and self-paced online sessions | - **Very good** | - **Good** | - **Average** | - **Poor** | - **Very Poor** |
| --- | --- | --- | --- | --- | --- |
| Discussions and being part with the group who also studied the same course | - **Very good** | - **Good** | - **Average** | - **Poor** | - **Very Poor** |
| Assignments | - **Very good** | - **Good** | - **Average** | - **Poor** | - **Very Poor** |
| Quizzes | - **Very good** | - **Good** | - **Average** | - **Poor** | - **Very Poor** |
| Announcements | - **Very good** | - **Good** | - **Average** | - **Poor** | - **Very Poor** |
| Case studies | - **Very good** | - **Good** | - **Average** | - **Poor** | - **Very Poor** |
| Working with experienced managers/mentors/ resource persons | - **Very good** | - **Good** | - **Average** | - **Poor** | - **Very Poor** |
| Learning by doing | - **Very good** | - **Good** | - **Average** | - **Poor** | - **Very Poor** |
| Attending face to face workshops and discuss some topics in depth | - **Very good** | - **Good** | - **Average** | - **Poor** | - **Very Poor** |

| **Question 17**   1. The most valuable skill / concept to me was: (please explain) | |
| --- | --- |
| 1. I wish more time was spent on the following skills / concepts: (please explain) | |
| 1. The least valuable skill / concept to me was: (please explain) | |
| 1. A topic or skill that was not addressed but that I needed / expected to learn in PHEP-BFE: (please explain) | |
| 1. What challenges, if any, did you have in meeting the requirements for your field work? |  |
| 1. What, if anything, would you have done differently had you been given more time? |  |
| 1. How relevant were the field work assignments to what you typically do as part of your job? |  |
| 1. Which aspects of the field work were most beneficial to your work? The least? |  |
| 1. Did anything come up in your field work that you wish you would have been better prepared for? |  |
| 1. Did Field Work meet your expectations in terms of quality of experience and learning? Please explain. |  |
